# Pilot Study Results from a Brief Intervention to Create Smoke-Free Homes

**DOI:** 10.1155/2012/951426

**Published:** 2012-05-17

**Authors:** Michelle C. Kegler, Cam Escoffery, Lucja Bundy, Carla J. Berg, Regine Haardörfer, Debbie Yembra, Gillian Schauer

**Affiliations:** ^1^Department of Behavioral Sciences and Health Education, Emory Prevention Research Center, Rollins School of Public Health, Emory University, 1518 Clifton Road NE, Atlanta, GA 30322, USA; ^2^Emory Prevention Research Center, Rollins School of Public Health, Emory University, 1518 Clifton Road NE, Atlanta, GA 30322, USA

## Abstract

Very few community-based intervention studies have examined how to effectively increase the adoption of smoke-free homes. A pilot study was conducted to test the feasibility, acceptability, and short-term outcomes of a brief, four-component intervention for promoting smoke-free home policies among low-income households. We recruited forty participants (20 smokers and 20 nonsmokers) to receive the intervention at two-week intervals. The design was a pretest-posttest with follow-up at two weeks after intervention. The primary outcome measure was self-reported presence of a total home smoking ban. At follow-up, 78% of participants reported having tried to establish a smoke-free rule in their home, with significantly more nonsmokers attempting a smoke-free home than smokers (*P* = .03). These attempts led to increased smoking restrictions, that is, going from no ban to a partial or total ban, or from a partial to a total ban, in 43% of the homes. At follow-up, 33% of the participants reported having made their home totally smoke-free. Additionally, smokers reported smoking fewer cigarettes per day. Results suggest that the intervention is promising and warrants a rigorous efficacy trial.

## 1. Introduction

Exposure to secondhand smoke (SHS) has a broad range of serious health consequences. SHS exposure increases the risk of lung cancer, stroke, and coronary heart disease [1–6]. Exposure to SHS can exacerbate asthma and underlying lung disease, contribute to respiratory problems, and reduce lung function in adults [5, 7]. Exposure is particularly dangerous to children, increasing the risk of respiratory infections, including asthma, bronchitis, and pneumonia, severity of asthma symptoms, middle ear infections, and sudden infant death syndrome [2, 8–13]. Risk for adverse health effects in children increases as the number of adult smokers in the household increases [2]. 

Due in large part to the increasing adoption of smoke-free environments in the USA, the home is currently a primary source of exposure to SHS for both children and nonsmoking adults [2]. The prevalence of smoke-free homes, defined as no smoking any place at any time, has increased rapidly in recent years [14]. These increases are associated with an expansion of smoke-free policies at the state and local level [14–16]. In 2008, an estimated 78% of homes in the USA were smoke-free [17]. However, rules that limit smoking in the home are less common in households in which at least one person smokes and in African American and low-income households [18–20].

Smoke-free homes have been shown to reduce exposure to SHS for both nonsmokers and children [12, 21–25]. Additionally, both longitudinal and cross-sectional studies show that smokers who have implemented smoke-free home rules are significantly more likely to make a quit attempt, be abstinent and smoke fewer cigarettes per day [19, 26–31]. Household smoking bans are also an important component of antismoking socialization and are linked to reduced likelihood of adolescent smoking [32, 33].

Few community-based intervention studies have examined how to effectively increase the adoption of smoke-free homes, particularly with the primary message focused on household smoking bans as opposed to smoking cessation [12]. Clinic-based interventions, often with a combined message of smoking cessation and reduced smoking in the home, have typically consisted of brief interventions with a verbal recommendation to reduce SHS exposure along with printed educational materials [12]. Home-based interventions have tended to be more intensive, usually involving 5–7 half-hour sessions over several months [12]. A review of home and clinic-based interventions reported mixed results in the clinic-based interventions and greater success in the more intensive home-based interventions [12]. However, little research has focused on brief and practical strategies for addressing SHS exposure through interventions focused explicitly on creating a smoke-free home [34]. Given the concentration of smoking in low-income households, the current study aimed to test the feasibility, acceptability, and short-term outcomes of a brief intervention for promoting smoke-free home policies among low-income households. We hoped to learn if smoking and nonsmoking members of low-income households would be interested in participating, whether they would participate in the full intervention and whether the intervention would motivate them to take steps to create a smoke-free home. We were also interested in their feedback on the intervention and suggestions for improvement.

## 2. Methods

### 2.1. Sample and Recruitment

We recruited participants from a county health department clinic in the metro Atlanta area. Participants were recruited in person by research staff and through fliers posted at the health department. Interested participants called our research office and were screened for eligibility. Eligible participants had to be 18 years or older, speak and understand English, be a smoker living with at least one other person in the household or a nonsmoker living with a smoker, and not have a total smoking ban. Only one participant per household was eligible. Approximately 300 fliers were distributed and 91 participants called the study office to express interest in participating. The study purpose and procedures were explained to eligible participants (21 were ineligible) and the first 20 smokers and first 20 non-smokers who agreed to participate provided verbal consent over the telephone and were enrolled (*n* = 40). Thirty-six participants completed the entire study.

### 2.2. Description of the Intervention

**Figure 1:**
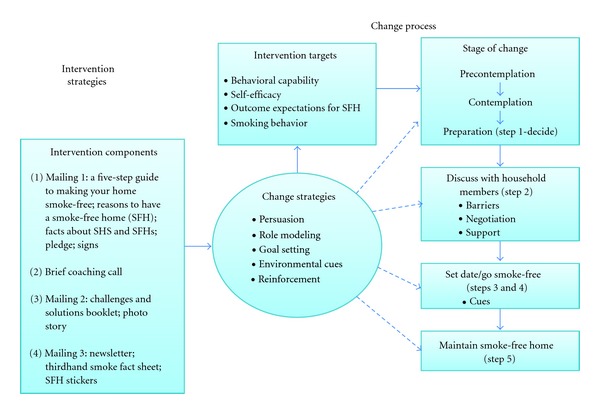
Model of behavior change: brief intervention to create smoke-free home policies in low-income households.

The smoke-free homes intervention consisted of four components: three mailings of print materials and one coaching call, aimed at increasing household smoking bans and reducing secondhand smoke exposure. The materials were designed to target both smokers and nonsmokers who allow smoking in the home. The conceptual model (Figure 1) is based on social cognitive theory [35–37] and the transtheoretical model's stages of change [38–40]. Social cognitive theory was selected because of its emphasis on both cognitive and environmental determinants of behavior and the interplay between them known as reciprocal determinism [37]. The intervention targets proximal determinants of behavioral capacity, self-efficacy, and outcome expectations related to creating a smoke-free home and smoking behaviors. Although not well studied with respect to smoke-free homes, these variables have been shown as important in a wide range of behavioral interventions based on social cognitive theory [37]. Through the use of persuasion, role modeling, goal setting, environmental cues and reinforcement—change strategies tied to social cognitive theory—participants were encouraged to work through the five steps of creating a smoke-free home. These include (1) deciding to create a smoke-free home, (2) talking to household members about making a home smoke-free, (3) setting a date for going smoke-free, (4) actually making a home smoke-free, and (5) keeping the home smoke-free. Because the five steps aligned quite well with stages of change as articulated in the transtheoretical model, we also included stages of change in the conceptual model [38]. This allowed us to focus the coaching component of the intervention on the appropriate step (or stage) for each participant.

The five steps emerged from our prior qualitative work on creating smoke-free homes (e.g., factors influencing the decision to go smoke-free, the need to talk to household members about a possible rule, challenges in enforcing the rule), combined with existing smoke-free home campaigns by the U.S. Environmental Protection Agency and Health Canada [41, 42]. Our earlier formative work on smoke-free homes included qualitative interviews with 102 households in rural Georgia with varying degrees of household smoking restrictions [43, 44]. Briefly, this work documented that family discussions about smoking bans focused heavily on protecting children. In homes with at least one nonsmoker, the smell and dangers of secondhand smoke and an aversion to breathing smoke were also frequently discussed. Conversations about a smoke-free home were usually initiated by women and/or nonsmokers. Conflict over the issue was rare, although challenges with enforcement and compliance were described by some participants [43, 44]. This formative research helped us develop intervention messages, for example, on common reasons to create a smoke-free home. Participant ideas for promoting a smoke-free home, which included environmental strategies such as posting no smoking signs in the home, helping the smoker find a comfortable place outside to smoke, and removing ashtrays and lighters, were also included in the educational materials. Finally, we asked about barriers to enforcing a ban. These barriers, such as feeling uncomfortable or concern over showing disrespect to a visitor or older relative were acknowledged in the materials as well, along with potential solutions.

All print materials were designed around the theme of “Some Things are Better Outside.” The first component, mailed after completion of the baseline survey, was a “tool-kit” for creating a smoke-free home. The tool-kit included a “*Five-Step Guide to a Smoke-Free Home” *which described the steps, tips, and strategies to plan for, make, and keep a smoke-free home. The guide was packaged in a 9′′ × 12′′ mailer that folds out to 18′′ × 24′′ when opened. The mailer was designed to be interactive and educational. It included definitions of secondhand smoke and smoke-free homes, a list of reasons to have a smoke-free home, truths about secondhand smoke, a tear-off pledge participants and household members could sign after deciding to make their home smoke-free, and two tear-off smoke-free home signs with adhesive tape strips.

The second component of the intervention was a coaching call. The coaching script incorporated the five steps as described in the “*Five-Step Guide to a Smoke-Free Home.” *The semistructured script elicited responses on the progress of making the home smoke-free, benefits of a smoke-free home, and challenges and barriers to setting a smoke-free home rule. A stage of change assessment was performed (i.e., have no interest in making home smoke-free, are thinking about making home smoke-free, decided to make home smoke-free, or already have a smoke-free home) to prompt the coach to provide stage-based messages. The coaching session ended with a summary of the call and goals for making and/or keeping a smoke-free home.

The third component included additional educational information in the form of a photo story which depicted a household comprising a mother, grandmother, and a child going through the process of making their home smoke-free. It provided information on secondhand smoke and its dangers, tips on having a conversation with the smoker in the home, ways to make smoking outside easier, and wants to celebrate being smoke-free. Also included in this mailing was a “*Challenges and Solutions: Keeping your Home Smoke-Free” *booklet. It provided ten commonly reported challenges derived from our formative research (e.g., you are not the head of the household and you cannot make the rules in your home; you live in an apartment and there is no porch or yard to use as a smoking area, etc.) and offered easy-to-implement solutions.

The fourth component included a newsletter with testimonials and success stories portraying families and their reasons for having a smoke-free home, as well as examples of ways to keep their home smoke-free. This mailing also included a thirdhand smoke fact sheet and six smoke-free home stickers that could be used as reminders to smoke outside (i.e., placed on bathroom mirrors, cigarette packs, ashtrays, etc.).

In addition to the formative research described above, we pretested our intervention materials, including the “Some Things are Better Outside” theme, through six focus groups (3 smokers and 3 non-smokers). Participants gave us feedback about each intervention component, and overall, had very positive comments about the intervention components (e.g., 5-step guide, pledge, signs, stickers). We also learned that there was little knowledge about thirdhand smoke, which prompted the inclusions of a small educational piece on this concept.

### 2.3. Procedures

We used a simple pretest posttest design with follow-up at two weeks after the intervention. After enrollment, participants were asked to complete a baseline survey by telephone which lasted approximately 30–45 minutes. All participants received the four intervention components at two-week intervals. Intervention components included three mailings and one coaching call. The first set of print materials was mailed after completion of the baseline survey, followed by a coaching call at week two, with the remaining print materials mailed at weeks four and six. A follow-up survey was conducted eight weeks after baseline. Each participant was compensated with a $25 gift card for completing each follow-up survey. Telephone surveys and coaching sessions were recorded for quality assurance. The study protocol was approved by the Emory University Institutional Review Board.

### 2.4. Measures

The baseline survey included questions related to smoking history, secondhand smoke exposure, cigarette consumption, cessation attempts, household composition and smoking status, beliefs about secondhand smoke, stage of readiness to restrict smoking in the home, self-efficacy to restrict smoking in the home, prior smoke-free home attempts, and secondhand smoke reduction behaviors.

#### 2.4.1. Process Evaluation

Process measures were collected at the eight-week follow-up to assess the receipt of mailed materials, the proportion of materials read, the usefulness and relevance of materials, satisfaction with the coaching session, and utilization of intervention materials such as posting signs, signing and/or posting the pledge, coming up with a list of reasons for making the home smoke-free, having the family talk, or calling the smoking cessation quitline telephone number provided in the materials.

#### 2.4.2. Outcome Measures (Primary)

**Smoke-Free Home Ban**

The primary outcome measure was self-reported presence of a total home smoking ban and was assessed at baseline and again through the 8-week follow-up survey using the item, “*Which statement best describes the rules about smoking inside your home?”* Participants were asked to select one of the following response options: *smoking is not allowed anywhere inside your home; smoking is allowed in some places or at some times; smoking is allowed anywhere inside your home; there are no rules about smoking inside your home *[45].

**Prior Smoke-Free Home Attempts**

We examined smoke-free home attempts by asking, “*In the last two months, has anyone tried to establish a smoke-free rule in your current home? By smoke-free, we mean that smoking is not allowed at any time or any place within your home”* [46].

**Secondhand Smoke Exposure**

Secondhand smoke exposure was measured using two items: *“How often does anyone smoke inside your home?” *with response options ranging from *daily* to *never *and *“During the past 7 days, how many days have people smoked in your home in your presence?”* [47].

#### 2.4.3. Outcome Measures (Secondary)

Three of our secondary outcomes were asked of smokers only: stage of change for quitting, cessation attempts, and number of cigarettes smoked per day.

**Stage of Change for Quitting Smoking**

Participants self-reported their smoking status both at baseline and at follow-up. Readiness to quit smoking among those who reported either “everyday” or “some days” of smoking at baseline was assessed using two additional items adapted from Velicer et al. [48]. In a yes/no format, we asked participants at baseline and eight-week follow-up, *“Are you thinking about quitting smoking within the next six months/30 days?” *

**Cessation Attempts**

Occurrence of quit attempts was assessed using the item, *“How many times during the past 2 months have you stopped smoking for more than one day because you were trying to quit smoking?”* adapted from the Behavioral Risk Factor Surveillance System [45].

**Cigarette Consumption**

One item was used to assess cigarette consumption per day, *“On average, on the days you smoke, how many cigarettes do you smoke in a day?”* [31].

**Smoking Restrictions in Cars**

An item adapted from Norman et al. [20] was used to assess smoking restrictions in cars. Participants were asked *“Now, what about smoking in your car or cars, would you say…” *and were provided the following response options: *there are no rules about smoking in the cars; smoking is sometimes allowed in some cars; smoking is never allowed in any car; there is no car. *

**Demographics**

Demographic information on the participant's ethnicity/race, age, gender, educational level, marital status, household income, and employment status was collected at baseline. Measures were adapted from the 2005 Behavioral Risk Factor Surveillance System [49].

### 2.5. Data Analysis

Results for primary and secondary outcomes were summarized using simple descriptive statistics including arithmetic mean, standard deviation, and percentage. Process evaluation measures were tested for differences in responses between smokers and nonsmokers with paired samples *t*-tests, Wilcoxon Mann Whitney tests, chi-squared tests of independence, and Fisher's exact tests depending on the nature of data collected. Changes in outcomes between baseline and follow-up were evaluated across all participants, as well as among smokers and non-smokers living with a smoker, using paired *t*-tests for continuous variables and the Wilcoxon signed rank sum test for ordinal variables. SPSS and SAS 9.3 were used to conduct descriptive as well as inferential analyses.

## 3. Results

### 3.1. Description of Study Participants

Most of the participants were African American (95%), and the majority were women (70%) (Table 1). Study participants had varying degrees of education, but none of the participants had completed college. Most participants were unemployed (65%), and of the 35% employed, less than half were employed full time. A large percentage (35%) of participants reported an annual household income of $10,000 or less and 58% lived with children under the age of 18. One quarter of the participants had no health care coverage, and 45% received Medicaid or Medical Assistance. Most homes (80%) were rented. The majority of participants (58%) lived in single-unit or detached homes, but 35% lived in an apartment, a condominium, or a multiunit complex.

### 3.2. Process Evaluation

Table 2 shows selected process evaluation findings by smoking status of the participant. A majority of participants read most or all of the materials (75%), with no significant difference between smokers and nonsmokers. Most participants (86%) reviewed the materials sometimes or even often, with smokers looking at the materials more than non-smokers (*P* = .03). In addition, participants found the materials relevant and useful. Notably, 89% reported the materials were very relevant and 95% reported they were very useful, with no differences by smoking status. Most liked the *5-Step Guidebook* best and did not like any of the materials least. Of the 36 participants who completed the follow-up survey, 81% came up with a list of reasons for making the home smoke-free and 97% had a talk with their family about making the home smoke-free. In addition, five participants (14%) reported calling smoking cessation services for support in quitting smoking. More than half (53%) signed the smoke-free home pledge, and more than 60% of participants posted the pledge, put up the signs, and used the stickers. A large majority of participants reported that the coaching call was very relevant to them (88%) and provided very useful information (85%), again with no significant differences by smoking status (not shown). General satisfaction with the call was high (94% very satisfied). Across all process measures, the smokers were as or more engaged with the intervention materials than the non-smokers. 

### 3.3. Primary Outcomes

Table 3 reports the impact of the intervention on smoking in the participants' homes. At follow-up, 78% of participants reported having tried to establish a smoke-free rule in their home, with significantly more non-smokers (94%) attempting a smoke-free home than smokers (63%) (*P* = .03). These attempts led to increased smoking restrictions, that is, going from no ban to a partial or total ban, or from a partial to a total ban, in 43% of the homes. At follow-up, 33% of the participants reported having made their home smoke-free (*P* < .0001), including 32% of smokers (*P* < .04) and 35% of nonsmokers (*P* < .004). The improvement in the smoke-free home status also resulted in a significant reduction of days on which smoking occurred in the home in the past week. Mean days of smoking in the home during the past week decreased from 5.3 days (SD = 2.4) to 2.6 days (SD = 2.7) (*P* < .0001). 

### 3.4. Secondary Outcomes

Smokers (*n* = 20) showed a significant improvement in readiness to quit smoking as assessed by the stages of change model (Table 4). At baseline, 35% planned to quit in the next 30 days and at follow-up 58% were in the preparation stage (*P* < .01). While only one individual reported quitting, 65% of the participating smokers reported at least two quit attempts during the two prior months. Moreover, cigarette consumption decreased significantly over the same time period, from 10.2 to 6.9 cigarettes per day (*P* < .04). Participation in this study also prompted participants to change their rules regarding smoking in the car. Among those with cars (*n* = 26), the proportion of smoke-free cars went from 20.0% to 38.9%, a significant increase (*P* < .005). 

## 4. Discussion

Assisting low-income households to go smoke-free has the potential to reduce exposure to SHS, help smokers to quit, and potentially disrupt the smoking initiation process in children and adolescents [31–33]. This study examined the feasibility, acceptability, and short-term outcomes of a brief intervention that explicitly targeted the creation of smoke-free homes. Results were promising for both smokers and non-smokers. We had no difficulty recruiting for the study, and retention was high. Participants reported high levels of interaction with the intervention materials and felt they were both relevant and helpful. Moreover, a relatively large percentage of participants engaged in the actions recommended through the intervention, such as talking with household members about going smoke-free and posting no-smoking signs. 

Short-term outcomes were promising, with about 1/3 of participants creating total smoking bans and over 40% tightening their household smoking restrictions in some way. These results are comparable or better than those from many intensive counseling interventions [12, 50]. A review of home and clinic-based interventions to reduce SHS exposure in the home reported an average effect size of  .34 [12]. A more recent review of interventions to create smoke-free homes during pregnancy or the neonatal period, typically based on counseling, was inconclusive due to poor study quality and the heterogeneity of outcomes reported [50]. Allmark and colleagues [51] reported evaluation results from an intervention similar to the one reported here, in which families who signed up for the program received a booklet and support materials. Although limited by no comparison group and a modest response rate, they found that among households that permitted some smoking at home before the initiative, about 78% became smoke-free after receiving the intervention program. 

Given the short follow-up period in our pilot study, we are uncertain whether participants will maintain their smoke-free homes. Even if long-term maintenance of smoke-free homes decreases to 10%, however, because of the ease of intervention delivery, this intervention has the potential to have a significant impact if widely disseminated. Most interventions to date have involved a more intensive counseling protocol; additional research is needed to establish whether brief interventions may be effective [12, 34, 50, 52]. Our next step is to conduct a randomized controlled trial of this brief intervention with follow-up at six months. 

There are several limitations to this study. This was a pilot study to test the feasibility a brief intervention of mailed print materials and coaching on making homes smoke-free. We evaluated the effectiveness of our intervention with only 40 families using a nonexperimental design; there was no control group. It is possible that social desirability, reactivity to the survey questions about smoke-free homes and/or other external factors are responsible for the positive outcomes. In addition, the sample for this study was predominantly urban, African American, and low income. The results may not be generalizable to other populations. The study also had a short follow-up period; future studies should examine the extent of relapse in home smoking bans. Finally, these data on home smoking bans were based on self-report and may not accurately reflect actual rules about smoking in the home. Future studies should use air nicotine monitors or other objective measures to validate self-reports of smoke-free homes. 

## 5. Conclusions

Results from the pilot study found that a brief educational intervention with families can increase smoke-free home policies and lower exposure to smoking in the home. In addition, this preliminary study suggests that the intervention can also help smokers reduce the number of cigarettes they smoke. Further research is needed to rigorously test the effectiveness of this brief intervention for increasing smoke-free homes, as well as its effects on other populations. We plan to conduct efficacy and effectiveness trials with large samples of low-income populations in several states. Because the home is a substantial source of SHS for children and nonsmoking adults, strategies to successfully eliminate exposure to smoke indoors are needed. Evaluating community-level interventions to create smoke-free homes can greatly reduce the impact of secondhand smoke on children and nonsmoking adults. 

## Figures and Tables

**Table 1:** Demographics of enrolled study participants.

Age	(*n* = 40)
18–39	38%
40–49	38%
50–60	25%
Race	
White	3%
African American	95%
Other	3%
Gender	
Female	70%
Education	
Less than high school	3%
Some high school	28%
High school graduate or GED	33%
Vocational or technical school	8%
Some college	30%
Employment status	
Employed	35%
Unemployed	65%
Annual household income	
$10,000 or less	35%
$10,001 to $15,000	13%
$15,001 to $20,000	18%
$20,001 to $25,000	13%
More than $25,000	18%
Home ownership	
Own	18%
Rent	80%
Other	3%
Type of housing	
Single-unit/detached home	58%
Townhome/duplex	8%
Apartment/condo/multiunit	35%
Number of children in the home	
None	43%
1	15%
2	15%
3	15%
4 or more	13%
Health care coverage	
No health care coverage	25%
Coverage through employer	18%
Medicaid or medical assistance	45%
Military (CHAMPUS, TIRCARE, or VA)	5%
Other	10%

**Table 2:** Process evaluation results for smoke-free home intervention.

	Total		Smokers		Non smokers		
	*N*	%	*N*	%	*N*	%	*P* value
	*N* = 36		*N* = 19		*N* = 17		
How much of the 1st mailing did you read? The mailing includes the 5-step guide to making your home smoke-free.							
Did not read any of it	1	3%	—	—	1	6%	
Read some of it	8	22%	4	21%	4	24%	
Read most of it	5	14%	1	5%	4	24%	.16
Read all of it	22	61%	14	74%	8	47%	
How often do you review/look at the materials?							
Never	2	6%	—	—	2	12%	
Rarely	3	8%	1	5%	2	12%	.03
Sometimes	19	53%	9	47%	10	59%	
Often	12	33%	9	47%	3	18%	
How relevant were the materials to you personally?							
Not at all	—	—	—	—	—	—	
A little	3	8%	1	5%	2	12%	
Somewhat	1	3%	1	5%	—	—	.81
Very/a lot	32	89%	17	89%	15	88%	
How useful or helpful was the information in the materials?							
Not at all	—	—	—	—	—	—	
A little	2	6%	1	5%	1	6%	
Somewhat	—	—	—	—	—	—	.99
Very/A lot	34	95%	18	95%	16	94%	
Did you (or someone in your home) any of the following? “Yes” reported.							
…come up with a list of reasons for making your home smoke-free?	29	81%	15	79%	14	82%	1.00
…have a talk with your family or household members about making your home smoke-free?	35	97%	18	95%	17	100%	1.00
…sign the pledge?	19	53%	14	74%	5	29%	.008
…post the pledge?	23	64%	13	68%	10	59%	.55
…put up the signs?	24	67%	14	74%	10	59%	.30
…use the stickers?	25	69%	17	90%	8	47%	.005
…call smoking cessation services?	5	14%	3	16%	2	12%	1.00

**Table 3:** Intervention impact on smoking rules in the home.

	All participants			Smokers			Non-Smokers		
	Baseline	Follow-up	*P* value	Baseline	Follow-up	*P* value	Baseline	Follow-up	*P* value
	*N* = 40	*N* = 36		*N* = 20	*N* = 19		*N* = 20	*N* = 17	
Smoking ban inside home									
Total ban	—	33%		—	32%		—	35%	
Partial ban	70%	58%	.0001	75%	58%	.04	65%	59%	.004
No ban	30%	8%		25%	11%		35%	6%	
Improvement in SFH status	N/A	43%		N/A	40%		N/A	45%	
SFH attempts	N/A	78%		N/A	63%		N/A	94%	
Smoking inside the home									
Daily	83%	53%		75%	53%		90%	53%	
Weekly	13%	14%		20%	11%		5%	18%	
Monthly	3%	6%	.0015	—	11%	.06	5%	—	.02
Less than monthly	3%	11%		5%	11%		—	12%	
Never	—	17%		—	16%		—	18%	
	Mean (SD)	Mean (SD)		Mean (SD)	Mean (SD)		Mean (SD)	Mean (SD)	
Days smoking occurred in the home last week	5.3 (2.4)	2.6 (2.7)	<.0001	5.4 (2.5)	1.8 (2.6)	<.0001	5.2 (2.4)	2.7 (2.8)	.002

**Table 4:** Intervention impact on smoking behaviors and stage of change for quitting.

	Baseline (*n* = 20)	Follow-up (*n* = 19)	*P* value
Stages of Change: quitting smoking			
Precontemplation	20%	5%	
Contemplation	45%	32%	0.01
Preparation	35%	58%	
Action	—	5%	
Smokers with quit attempts	N/A	65%	
	Mean (SD)	Mean (SD)	
Cigarettes per day	10.2 (5.7)	6.9 (6.0)	0.04

## References

[1] Hackshaw AK, Law MR, Wald NJ (1997). The accumulated evidence on lung cancer and environmental tobacco smoke. *British Medical Journal*.

[2] United States Department of Health and Human Services (2006). *The Health Consequences of Involuntary Exposure to Tobacco Smoke: A Report of the Surgeon General*.

[3] Taylor R, Najafi F, Dobson A (2007). Meta-analysis of studies of passive smoking and lung cancer: effects of study type and continent. *International Journal of Epidemiology*.

[4] Zhang X, Xiao OS, Yang G (2005). Association of passive smoking by husbands with prevalence of stroke among Chinese women nonsmokers. *American Journal of Epidemiology*.

[5] International Agency for Research on Cancer (IARC) (2009). *Evaluating the Effectiveness of Smoke-Free Policies, in Handbooks of Cancer Prevention, Tobacco Control*.

[6] Law MR, Morris JK, Wald NJ (1997). Environmental tobacco smoke exposure and ischaemic heart disease: an evaluation of the evidence. *British Medical Journal*.

[7] Eisner MD, Yelin EH, Henke J, Shiboski SC, Blanc PD (1998). Environmental tobacco smoke and adult asthma the impact of changing exposure status on health outcomes. *American Journal of Respiratory and Critical Care Medicine*.

[8] United States Environmental Protection Agency, Office of Health Environmental Assessment, and Office of Research and Development (1992). *Respiratory Health Effects of Passive Smoking: Lung Cancer and Other Disorders*.

[9] DiFranza JR, Lew RA (1996). Morbidity and mortality in children associated with the use of tobacco products by other people. *Pediatrics*.

[10] Anderson HR, Cook DG (1997). Passive smoking and sudden infant death syndrome: review of the epidemiological evidence. *Thorax*.

[11] McMartin KI, Platt MS, Hackman R (2002). Lung tissue concentrations of nicotine in sudden infant death syndrome (SIDS). *Journal of Pediatrics*.

[12] Gehrman CA, Hovell MF (2003). Protecting children from environmental tobacco smoke (ETS) exposure: a critical review. *Nicotine and Tobacco Research*.

[13] California Environmental Protection Agency (2005). *Air Resources Board. Proposed Identification of Environmental Tobacco Smoke as a Toxic Air Contaminant*.

[14] Zhang X, Martinez-Donate AP, Kuo D, Jones NR, Palmersheim KA (2012). Trends in home smoking bans in the USA, 1995–2007: prevalence, discrepancies and disparities. *Tobacco Control*.

[15] Cheng KW, Glantz S, Lightwood JM (2011). Association between smokefree laws and voluntary smokefree-home rules. *American Journal of Preventive Medicine*.

[16]  Hovell MF, Lessov-Schlaggar C, Ding D (2011). Smokefree community policies promote home smoking bans unknown mechanisms and opportunities for preventive medicine. *American Journal of Preventive Medicine*.

[17] Centers for Disease Control and Prevention (2009). State-specific secondhand smoke exposure and current cigarette smoking among adults—United States, 2008. *Morbidity and Mortality Weekly Report*.

[18] Koepke D, Flay B, Johnson C (1990). Health behaviors in minority families: the case of cigarette smoking. *Family and Community Health*.

[19] Gilpin EA, White MM, Farkas AJ, Pierce JP (1999). Home smoking restrictions: which smokers have them and how they are associated with smoking behavior. *Nicotine and Tobacco Research*.

[20] Norman GJ, Ribisl KM, Howard-Pitney B, Howard KA (1999). Smoking bans in the home and car: do those who really need them have them?. *Preventive Medicine*.

[21] Biener L, Cullen D, Di ZX, Hammond SK (1997). Household smoking restrictions and adolescents’ exposure to environmental tobacco smoke. *Preventive Medicine*.

[22] Wakefield M, Banham D, Martin J, Ruffin R, McCaul K, Badcock N (2000). Restrictions on smoking at home and urinary cotinine levels among children with asthma. *American Journal of Preventive Medicine*.

[23] Pizacani BA, Martin DP, Stark MJ, Koepsell TD, Thompson B, Diehr P (2003). Household smoking bans: which households have them and do they work?. *Preventive Medicine*.

[24] Brenner H, Mielck A (1992). Smoking prohibition in the workplace and smoking cessation in the Federal Republic of Germany. *Preventive Medicine*.

[25] Jeffery RW, Kelder SH, Forster JL, French SA, Lando HA, Baxter JE (1994). Restrictive smoking policies in the workplace: effects on smoking prevalence and cigarette consumption. *Preventive Medicine*.

[26] Mills AI, Messer K, Gilpin EA, Pierce JP (2009). The effect of smoke-free homes on adult smoking behavior: a review. *Nicotine and Tobacco Research*.

[27] Farkas AJ, Gilpin EA, Distefan JM, Pierce JP (1999). The effects of household and workplace smoking restrictions on quitting behaviours. *Tobacco Control*.

[28] Kegler MC, Malcoe LH (2002). Smoking restrictions in the home and car among rural Native American and White families with young children. *Preventive Medicine*.

[29] Okah FA, Choi WS, Okuyemi KS, Ahluwalia JS (2002). Effect of children on home smoking restriction by inner-city smokers. *Pediatrics*.

[30] Pizacani BA, Martin DP, Stark MJ, Koepsell TD, Thompson B, Diehr P (2004). A prospective study of household smoking bans and subsequent cessation related behaviour: the role of stage of change. *Tobacco Control*.

[31] Messer K, Mills AL, White MM, Pierce JP (2008). The Effect of Smoke-Free Homes on Smoking Behavior in the U.S.. *American Journal of Preventive Medicine*.

[32] Clark PI, Schooley MW, Pierce B, Schulman J, Hartman AM, Schmitt CL (2006). Impact of home smoking rules on smoking patterns among adolescents and young adults. *Preventing Chronic Disease*.

[33] Wakefield MA, Chaloupka FJ, Kaufman NJ, Orleans CT, Barker DC, Ruel EE (2000). Effect of restrictions on smoking at home, at school, and in public places on teenage smoking: cross sectional study. *British Medical Journal*.

[34] Hovell MF, Hughes SC (2009). The behavioral ecology of secondhand smoke exposure: a pathway to complete tobacco control. *Nicotine and Tobacco Research*.

[35] Bandura A (1986). *Social Foundations of Thought and Action: A Social Cognitive Theory*.

[36] Bandura A (1977). *Social Learning Theory*.

[37] McAlister A, Perry C, Parcel G, Glanz K, Rimer B, Viswinath K (2008). How individuals, environments and health behaviors interact. *Health Behavior and Health Education*.

[38] Prochaska J, Redding C, Evers K, Glanz K, Rimer B, Viswinath K (2008). The transtheoretical model and stages of change. *Health Behavior and Health Education*.

[39] Prochaska JO, DiClemente CC, Norcross JC (1992). In search of how people change: applications to addictive behaviors. *American Psychologist*.

[40] Prochaska JO, Velicer WF, DiClemente CC, Fava J (1988). Measuring processes of change: applications to the cessation of smoking. *Journal of Consulting and Clinical Psychology*.

[41] U.S. Environmental Protection Agency Smoke-Free Homes. http://www.epa.gov/smokefree/index.html.

[42] Health Canada (2006). *Make Your Home and Car Smoke-Free: A Guide to Protecting your Family from Second-Hand Smoke*.

[43] Kegler MC, Escoffery C, Groff A, Butler S, Foreman A (2007). A qualitative study of how families decide to adopt household smoking restrictions. *Family and Community Health*.

[44] Escoffery C, Kegler MC, Butler S (2009). Formative research on creating smoke-free homes in rural communities. *Health Education Research*.

[45] CDC

[46] Martínez-Donate AP, Hovell MF, Hofstetter CR (2005). Smoking, exposure to secondhand smoke, and smoking restrictions in Tijuana, Mexico. *Revista Panamericana de Salud Publica/Pan American Journal of Public Health*.

[47] World Health Organization *Tobacco Free Initiative: Surveillance and Monitoring*.

[48] Velicer WF, Hughes SL, Fava JL, Prochaska JO, DiClemente CC (1995). An empirical typology of subjects within stage of change. *Addictive Behaviors*.

[49] CDC http://www.cdc.gov/brfss/technical_infodata/surveydata/2005.htm.

[50] Baxter S, Blank L, Everson-Hock ES (2011). The effectiveness of interventions to establish smoke-free homes in pregnancy and in the neonatal period: a systematic review. *Health Education Research*.

[51] Allmark P (2012). Evaluation of the impact of a smoke-free home initiative in Rotherham, a deprived district in Northern England. *European Journal of Public Health*.

[52] Hovell MF, Wahlgren DR, Lile S (2011). Providing coaching and cotinine results to preteens to reduce their secondhand smoke exposure: a randomized trial. *Chest*.

